# Strategies to Address or Prevent Self-Neglect among Older Adults: A Systematic Review

**DOI:** 10.1093/geroni/igaf122.3144

**Published:** 2025-12-31

**Authors:** Zhiqi Yi, Lora Chehab, Joy Ernst, Fei Sun, Eleanor Smith

**Affiliations:** University of Kansas, Lawrence, Kansas, United States; Michigan State University, East Lansing, Michigan, United States; Wayne State University, Detroit, Michigan, United States; Michigan State University, East Lansing, Michigan, United States; University of Michigan, Ann Arbor, Michigan, United States

## Abstract

Self-neglect is a growing form of elder maltreatment characterized by one’s inability to perform essential self-care tasks due to physical or mental impairment or diminished capacity. Reports of self-neglect to Adult Protective Services far outnumber other types of maltreatment. While research has identified characteristics and risk factors for self-neglect, the best practices to address or prevent elder self-neglect remain unclear. An overview of theoretical models and practical approaches can enhance understanding and how to successfully intervene with older adults who self-neglect. A search of peer-reviewed literature between January 1990 and September 2024 across six databases (i.e., PsycINFO, PubMed, Cochrane Library, EMBASE, WOS, and MedLine) yielded twenty eligible articles with empirical, conceptual, and case research designs. Two reviewers independently screened, read, and rated the quality of all articles. Three authors independently identified themes using a thematic analysis approach and reached a consensus. Various intervention models and approaches with different focal points were identified. Approaches targeting older adults, their caregivers or both, used nutritional intervention, case management, and psychosocial intervention. Approaches targeting service professionals emphasized enhancing service techniques, multidisciplinary collaboration, and community support. Macro-level approaches stressed the need for changes in the community and social systems. These findings emphasized the complexities of self-neglect in older adults, the importance of helping techniques, and the need for an integrated and holistic approach. Although no single best practice is concluded, a holistic, multidisciplinary, and multilevel approach to self-neglect is recommended.

